# Information transfer via temporal convolution in nonlinear optics

**DOI:** 10.1038/s41598-020-72170-9

**Published:** 2020-09-11

**Authors:** Philippe Lassonde, Heide Ibrahim, Adrien Leblanc, José Azaña, Bruno E. Schmidt, François Légaré

**Affiliations:** 1 INRS-EMT, 1650 Blvd Lionel-Boulet, Varennes, QC J3X1S2 Canada; 2 Laboratoire d’Optique Appliquée, École polytechnique, ENSTA, CNRS, Université Paris Saclay, Palaiseau, France; 3 Few-Cycle Inc., 2890 rue de Beaurivage, Montreal, QC H1L5W5 Canada

**Keywords:** Nonlinear optics, Ultrafast photonics

## Abstract

Nonlinear parametric processes involving ultrashort pulses are typically carried out in *time domain*, which mathematically corresponds to a convolution of their frequency spectra. In contrast, this *spectral convolution* changes into a multiplication operation when performing the nonlinear interaction in *frequency domain*. Here, we extend the scope of frequency-domain nonlinear optics by demonstrating its ability to perform a *temporal convolution*. Through this approach, nonlinear optical operations that are inaccessible in time domain can be realised: specific optical information can be coherently advanced by picoseconds within a pulse sequence—a newly generated second harmonic pulse carries the amplitude and phase information of two input pulses. This central pulse is isolated when using an input field consisting of two cross-polarized input pulses in combination with type-II second harmonic generation. The effects of nonlinear temporal convolution can be viewed from the aspect of signal processing and pulse shaping, where the nonlinear interaction in the parametric crystal plays the role of a dynamic linear optical filter—in contrast to conventional static filters—with a shaping mask instantaneously adapting to the laser field.

## Introduction

Convolution is a ubiquitous process that serves to describe effects ranging from acoustic echoes to the blurring of photographic images. In optics and electrical engineering, many devices are described as linear time-invariant systems which are characterized by their impulse response: an output signal is obtained from the temporal convolution of an input signal with the impulse response of the system^1^. One example is a photodiode detecting an ultrashort laser pulse; the dynamic object function (*c.f.* a femtosecond laser pulse) is convolved with the much slower, quasi-static response function of the photodiode. Therefore, the output signal, i.e. the oscilloscope trace on a nanosecond level, is insensitive to the actual laser pulse temporal profile.

This situation of a dynamic signal convolved with a quasi-static response is also found in the case of programmable pulse shapers such as spatial light modulators or acousto-optic modulators^2,3^. While they can provide an adjustable impulse response to shape the temporal profile of ultrashort pulses, and even though its shaping mask can be refreshed up to hundred thousand times per second^4^, such filters are static compared to the timescale of the laser field.

The convolution with dynamic response functions on the other hand is rarely explored. For example a “space-to-time mapping processor” was introduced about 20 years ago^5^. Noticeably, this early work utilized cascaded multi-beam interactions in the frequency domain of a *4f.*-setup. In a related experiment, the spectral and phase information of a double pulse was symmetrically inverted by aid of two cascaded interactions with two independent reference pulses^6^. The input double pulse remained a double pulse at the output.

Here, we describe a nonlinear scheme that performs a direct temporal convolution of a single input beam in the absence of any external reference beams. Such operation follows the intuitive picture where the convolution of a double pulse leads to the generation of a central third pulse. We demonstrate experimentally that phase and amplitude information from the trailing pulse of the input beam are transferred to the preceding central pulse of the output sequence. This surprising linear information transfer between two femtosecond laser pulses separated in time is possible upon nonlinear processing in the frequency domain. Temporal convolution between two dynamic functions is the prerequisite for this unique result.

In general, frequency mixing nonlinear processes occurring in time-domain are described by a spectral convolution of $\stackrel{\sim }{E}\left(\omega \right)$, the frequency-domain representation of the input pulse field They give rise to numerous applications in nonlinear optics, including optical parametric amplification or difference frequency generation^7,8^, but also extracting the spectral phase of ultrashort pulses using frequency-resolved optical gating techniques^9,10^. All these examples have in common that they depend on frequency cross-talk which is given in a spectral convolution. That is, all frequency components are mixing during the presence of the laser pulse. In frequency-domain nonlinear optics, on the inverse, the spectral components of an ultrashort pulse are discretized and thus, do not interact with each other. For the case of frequency domain second harmonic generation (FSHG), this concept allowed for the linear transfer of spectral phases in nonlinear optics, and for pulse shaping of laser harmonics in the deep UV^11,12^.

## Results

**Figure 1:**
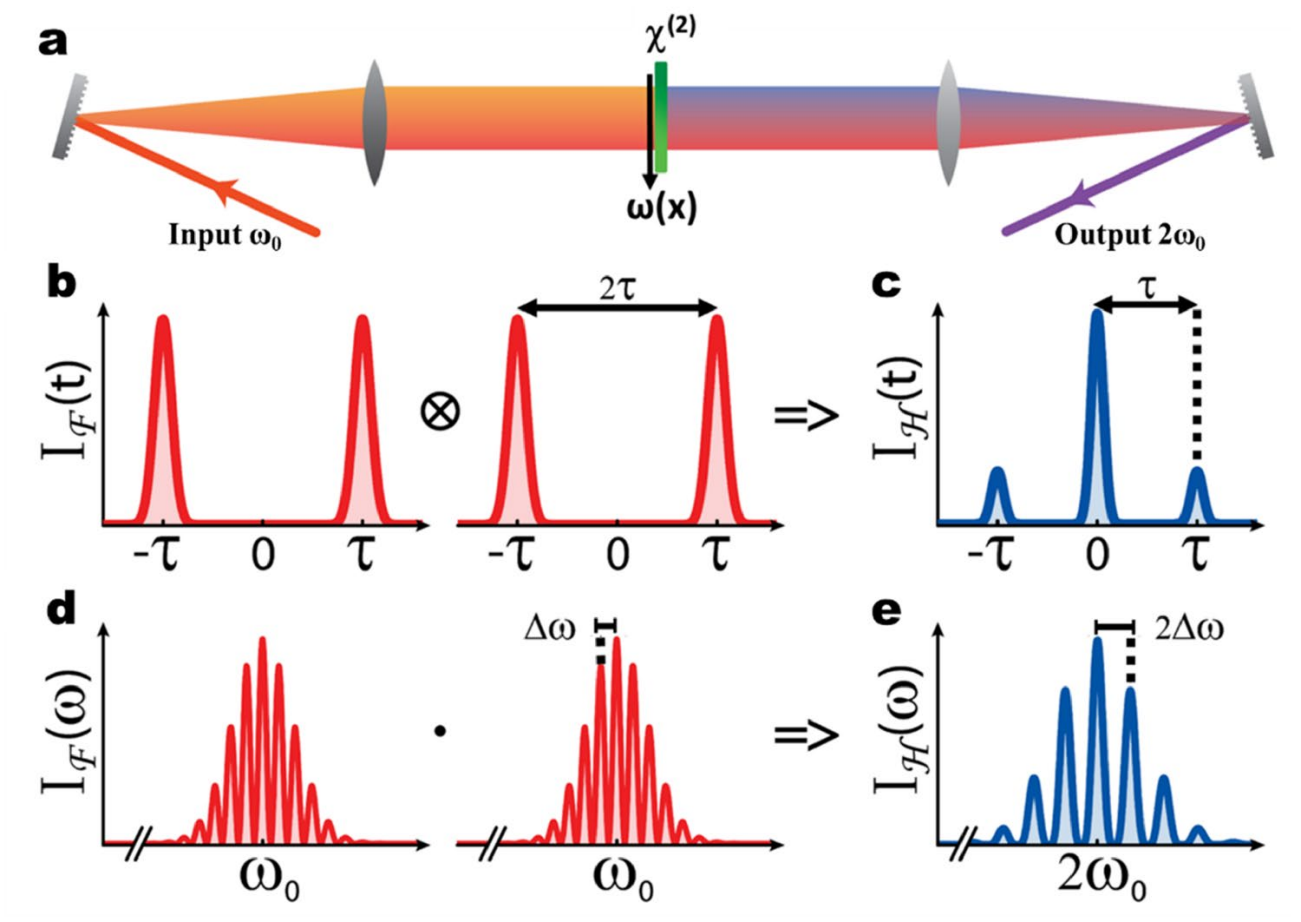
FSHG optical system. (**a**) The field is projected into the Fourier plane using a grating and a focusing lens separated by the focal distance. Through the χ^(2)^ interaction with the nonlinear crystal located in the Fourier plane (green line), a field at the second harmonic is generated. The total field is recombined after the second lens and grating. (**b**) The fundamental field entering the *4f*-setup consists of a pair of pulses having a relative delay $\Delta t=2\tau$. (**c**) From the temporal convolution of the fundamental pulse pair, three second harmonic pulses are generated at the output, separated by $\Delta t=\tau$. (**d**–**e**) In frequency, the corresponding spectra are characterized by interference modulations with twice the period at the central frequency $2{\omega }_{0}.$

The general layout of the exploited FSHG system is illustrated in Fig. 1a. It consists of a *4f.*-setup with a nonlinear crystal in the frequency plane^13–15^. In this plane, the spectrum is dispersed spatially along a focal line as in a spectrometer. The frequencies are separated within focussed narrow spectral slices.

As a consequence, there is no cross-talk between the spectral components, and the second harmonic field is given by^12^:

1 $$\stackrel{\sim }{\mathbf{E}}\left(2{\varvec{\upomega}}\right)\propto \stackrel{\sim }{\mathbf{E}}\left({\varvec{\upomega}}\right)\cdot \stackrel{\sim }{\mathbf{E}}\left({\varvec{\upomega}}\right)$$

This equation is equivalent to a temporal convolution:

2 $$\mathbf{E}\left(\frac{\mathbf{t}}{2}\right)\propto \mathbf{E}\left(\mathbf{t}\right)\otimes \mathbf{E}\left(\mathbf{t}\right)$$

In practice, this means that while spectral cross-talk is switched off, temporal cross-talk is enabled. This is in strong contrast to conventional time-domain SHG where the second harmonic field results from the product ${\varvec{E}}({\varvec{t}})\cdot {\varvec{E}}({\varvec{t}})$, so that temporal convolution operations cannot be accessed. To our knowledge, the operations of Eqs. (1) and (2) are unique to a spectral device employing optical nonlinearities in the Fourier domain.

Here, employing frequency domain nonlinear conversion, such temporal convolution of an optical field is demonstrated experimentally. To illustrate our concept, we consider two femtosecond pulses delayed by a time 2τ much longer than their respective durations, as shown in Fig. 1b. Adapting the concept of a temporal convolution, one would expect at the output of the FSHG setup the sequence of three distinct second harmonic pulses depicted in Fig. 1c, as we will discuss in detail later.

**Figure. 2:**
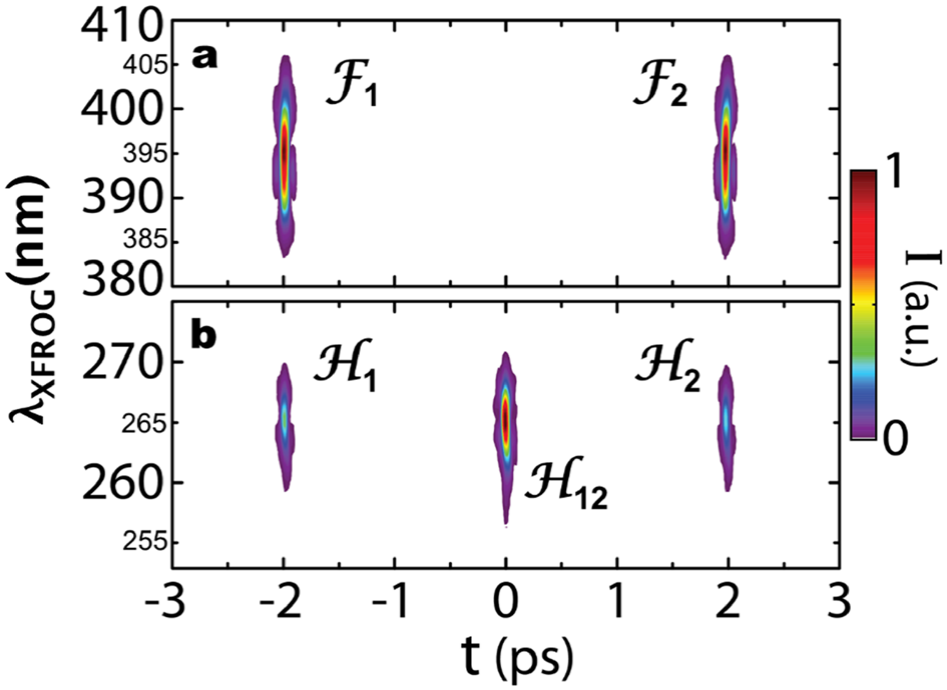
X-FROG spectrograms of the pulse sequence after the FSHG stage. (**a**) Pair of identical fundamental pulses with 35-fs duration, separated by 4 ps representing the input pulses; (**b**) Triplet of identical second-harmonic pulses measured with the same reference pulse. The FSHG pulses are spaced by 2 ps.

Experimentally, we initially consider a sequence of two identical fundamental pulses of 35-fs duration and separated by ~ 4 ps generated with a Michelson interferometer. They are frequency doubled by propagating through a FSHG setup after which all pulses (fundamental and second harmonic) are characterized through XFROG measurements obtained with a weak reference pulse at 790 nm^10^. This reference pulse has the effect of subtracting the average group delay of the overall system for all measurements. The XFROG spectrograms of all output pulses are shown in Fig. 2a for the fundamental (XFROG signal at 395 nm) and Fig. 2b for the second harmonic pulses (XFROG signal at 263 nm) coming out of the FSHG system. The central wavelength of the XFROG signal is determined from the sum-frequency generation process with the reference pulse at the fundamental central wavelength λ_0_ = 790 nm. Therefore, we have signals at λ_XFROG_ = λ_0_/2 = 395 nm and λ_XFROG_ = λ_0_/3 = 263 nm, for the fundamental and second-harmonic pulses, respectively. At 395 nm, the pair of fundamental pulses delayed by 4 ps is observed and each individual pulse is referred to as F1 and F2. At 263 nm, the spectrogram exhibits three pulses: An early pulse, referred to as H1 and leaving the system at the same time as F1, a second pulse H12, leaving the system 2 ps later, thus right in between F1 and F2, and finally, a third pulse H2 coincident with F2.

With a simple test, we validate the respective contributions from the fundamental pulses to the generated second-harmonic pulses. For instance, one arm of the Michelson interferometer is blocked to let only one fundamental pulse going through the system, either the pulse F1 or F2. It is observed that if F1 is blocked, the harmonic pulses H1 and H12 are suppressed, with H2 remaining. While if F2 is blocked, pulses H12 and H2 disappear and H1 remains. This test confirms that the harmonic pulse H12 is a cross-term originating from both F1 and F2. Inversely, H1 and H2 are non-cross terms respectively and exclusively originating from F1 and F2. Those experimental observations are presented in the Supplementary Information document.

**Figure 3:**
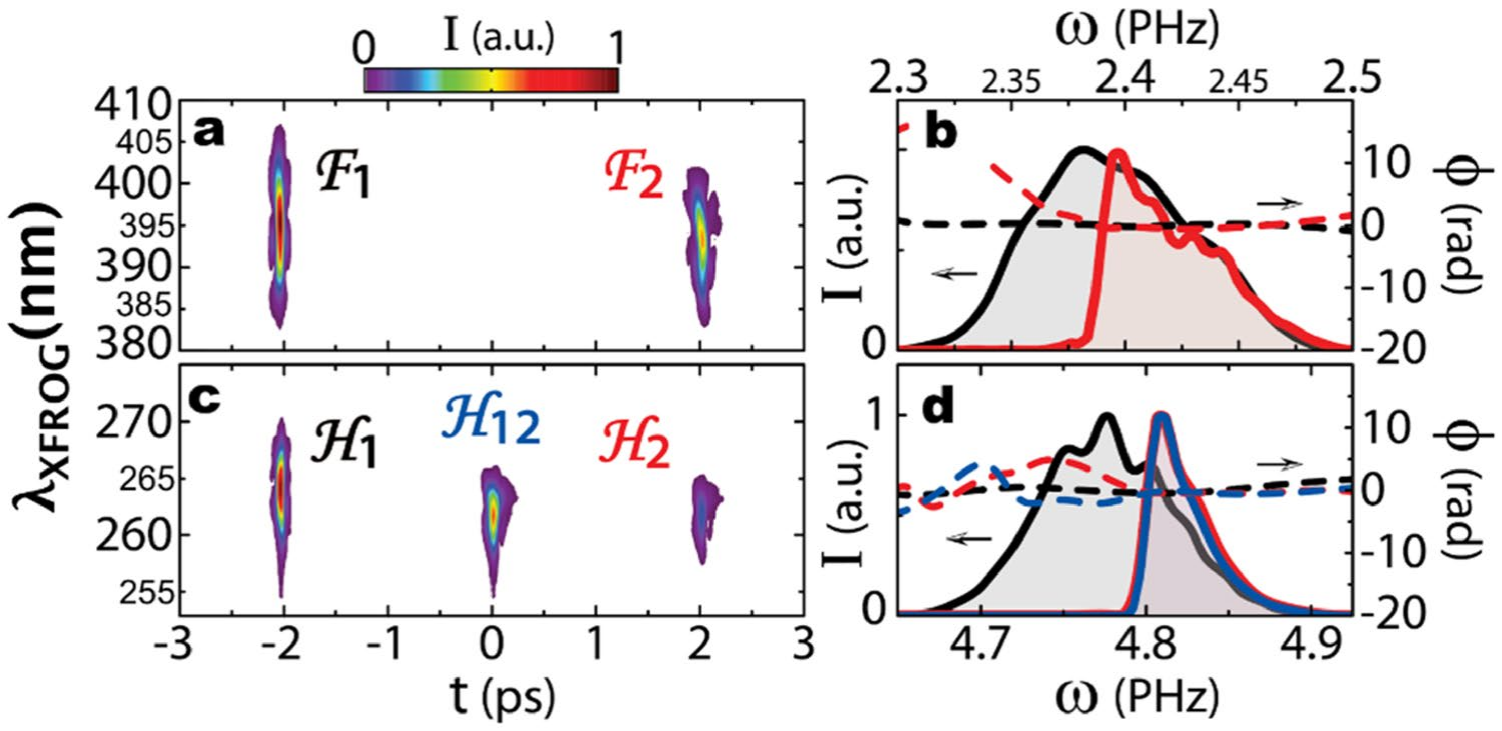
Coherent transfer of information within the laser field through interferometric FSHG. An amplitude filter is inserted in the longer Michelson arm before the nonlinear stage; XFROG spectrograms featuring the amplitude distribution among both pulse sequences: (**a**) fundamental pulses and (**c**) second-harmonic pulses. (**b**) Spectrum (solid line) and phase (dashed line) of individual pulses (black F1, red F2) retrieved from XFROG reconstruction. The spectrum of the delayed pulse at ω_0_ (F2) has a sharp cut-off. (**d**) The sharp cut-off is transferred from F2 at 2ω_0_ to two pulses at two different delays, H12 and H2. Notably, H12 is leaving the system 2 ps before F2.

**Figure 4:**
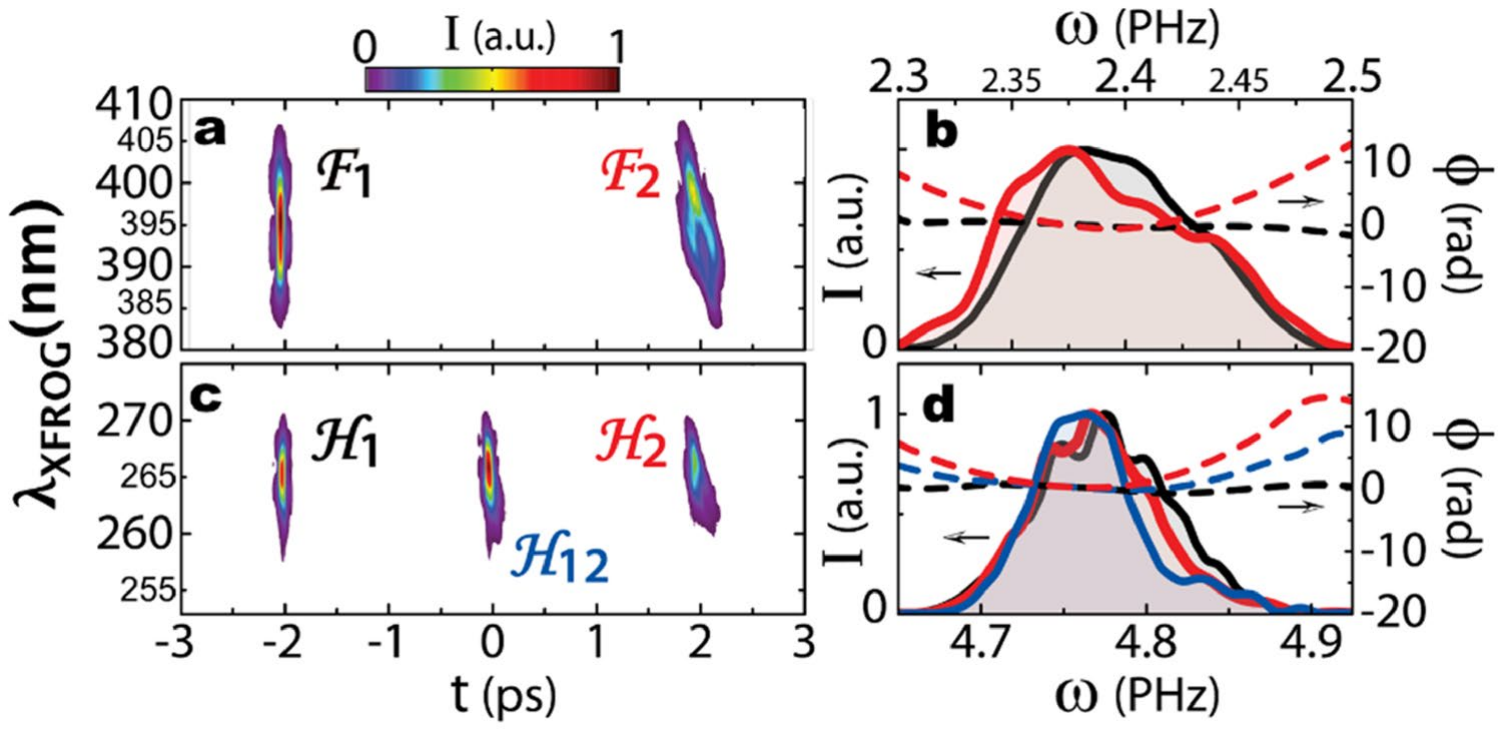
Coherent transfer of the phase through interferometric FSHG within the time window available for temporal convolution: A spectral phase filter (a 50 mm fused silica rod introducing positive group-delay dispersion) is inserted in the delayed Michelson arm before the nonlinear stage. Left column: XFROG spectrograms of the fundamental (**a**) and FSHG pulses (**c**) revealing the distribution of both pulse sequences at ω_0_ and 2ω_0_. (**b**) Spectrum (solid line) and phase (dashed line) of individual pulses (black F1, red F2) retrieved from XFROG reconstruction. The delayed pulse at ω_0_ (F2) has a positive chirp. (**d**) Its quadratic phase is transferred from F2 to the second harmonic pulses H12 and H2, where the phase stroke is doubled. H12 is leaving the system 2 ps before F2.

In a further step, the control of the cross-term pulse is demonstrated both in amplitude and phase by shaping the most delayed fundamental pulse F2. For instance, by inserting an optical element in the corresponding arm of the Michelson interferometer, F2 is modified either in spectral amplitude (Fig. 3) or in spectral phase (Fig. 4). In both cases, the relative delay between the two fundamental pulses is re-adjusted to ~ 4 ps after the insertion of the optical element.

The effect of those added optical components on the fundamental pulses is visible from the XFROG spectrograms, as F2 significantly differs from F1: in the case of the amplitude filter in Fig. 3a, a spectral filter (Semrock Inc.) is blocking any wavelength above 785 nm. In consequence, the corresponding XFROG signal of F2 is truncated compared to F1. In the situation of phase variation with a dispersive component consisting of a 50-mm long fused silica rod (Fig. 4a), a positive linear chirp is observed for F2. To quantify those variations, each individual pulse is retrieved with a XFROG reconstruction algorithm. In Fig. 3b, the retrieved spectrum of pulse F2 exhibits the sharp edge introduced by the amplitude filter (red solid line). In Fig. 4b, the retrieved spectral phase of F2 shows the positive quadratic phase introduced by the fused silica rod (red dashed line).

The second-harmonic pulse sequence after the amplitude and phase modifications of F2 is also characterized by XFROG. In each situation, the pulse H1 remains identical. For the amplitude filter, the sharp edge of the spectrum of F2 is transferred to both H12, the central pulse and to H2, the latest second-harmonic pulse, as observed in the spectrogram (Fig. 3c) and also in the retrieved pulses (Fig. 3d, blue and red solid lines). In the case of the dispersive component, the linear chirp of F2 is transferred in the same manner to both pulses H12 and H2, see Fig. 4c. Considering the retrieved phase in Fig. 4d, we observe a flat spectral phase for H1 (black dashed line) as expected, since F1 is transform-limited. In contrast, for H12 and H2, a quadratic phase is observed with the cross-term H12 having the phase of F2, and H2 having twice this phase. This transfer of linear chirp is visible directly from the XFROG spectrogram of Fig. 4c, with the slope of H2 being identical to that of F2 and twice that of H12. Combining these results admits the linear transfer of amplitude and phase information along the temporal axis of the second harmonic laser field.

In summary, in our experiments, we process two identical pulses delayed by several picoseconds and we obtain a sequence of three distinct second harmonic pulses. Then, by modifying the most delayed fundamental pulse, we observe the coherent transfer of information from the fundamental to the second harmonic pulses at different delays. As such, it is confirmed that one of the generated pulses is effectively the product of a temporal convolution of the initial field, carrying the amplitude and phase of both fundamental pulses.

**Figure 5:**
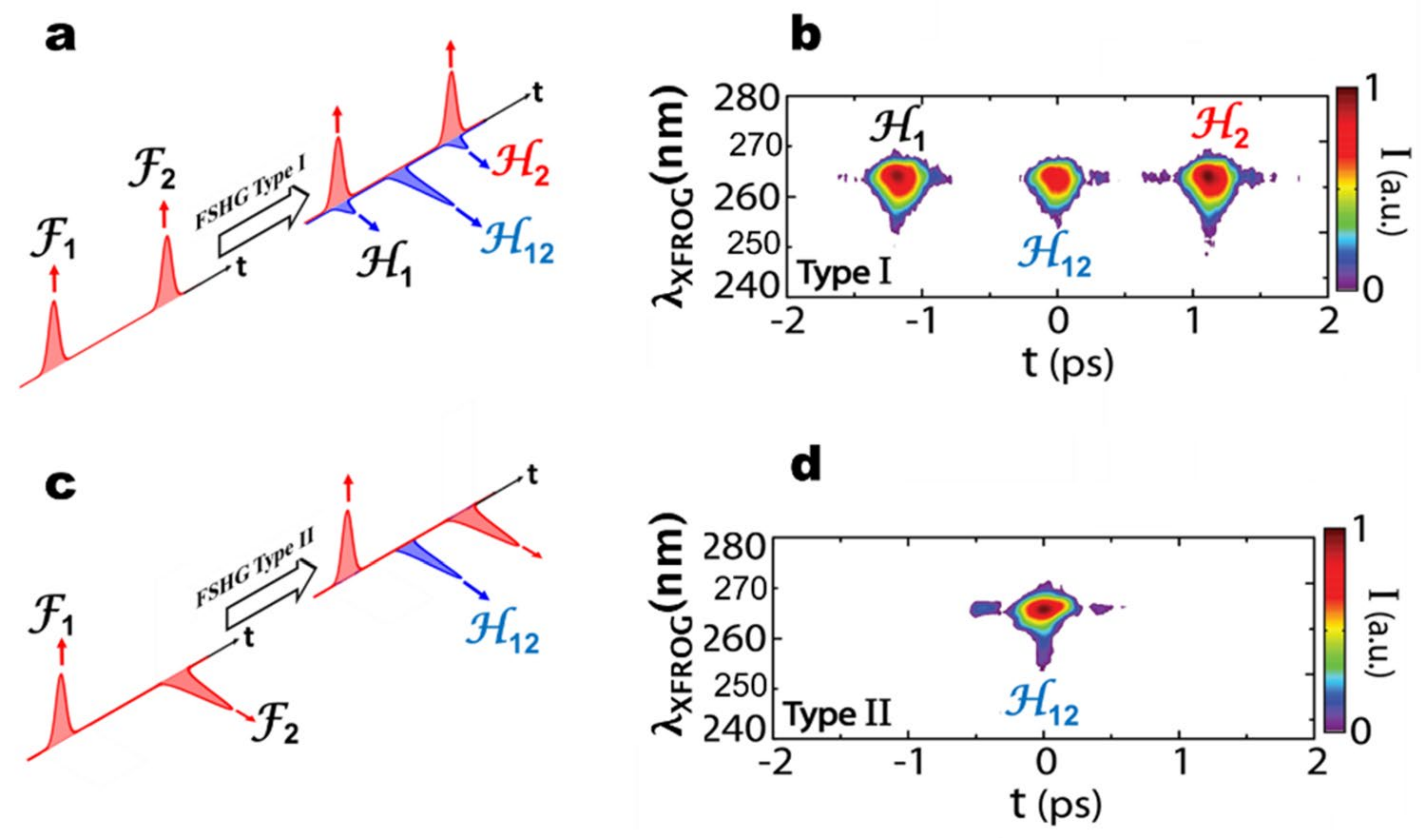
XFROG spectrograms of the pulse sequence obtained from FSHG of a pair of delayed pulses with parallel and orthogonal polarization states in type-I and type-II nonlinear crystals. (**a**) Both fundamental pulses have parallel polarization (arrows indicating directions of polarization). Three second harmonic pulses are generated in type I phase-matching, H1, H12 and H2. (**b**) Experimental FSHG pulses corresponding to (**a**). (**c**) One fundamental pulse has parallel polarization and the other, delayed, has orthogonal polarization. Using a type-II BBO crystal, phase-matching is possible only for orthogonal polarization states. This condition is fulfilled only for the cross-term H12 at the intermediate delay between the two fundamental pulses. (**d**) Experimental isolated FSHG pulse corresponding to (**c**).

In a last experiment, we demonstrate the ability to isolate the convolved term by controlling the polarizations and adapting the phase-matching conditions of the nonlinear interaction. For this, we consider frequency doubling two delayed pulses having orthogonal polarizations. For this specific experiment, the amplitude and phase of the harmonic pulses were not retrieved because the crystals involved were 700 µm thick and the phase-matching bandwidth was limited compared to the full bandwidth 35 fs pulses. We were interested here in the polarization transfer and the generated pulse sequence through the convolution operation. In this case, employing a type-II crystal, it is assumed that only the pulse H12 fulfills the phase-matching conditions. This situation is illustrated in Fig. 5c and the experimental result is confirmed in Fig. 5d, where only the pulse H12 is generated. In comparison, the situation of F1 and F2 with identical polarizations and type-I crystal is illustrated in Fig. 5a. The result with three pulses is shown in Fig. 5b with corresponding pulse delays (F1 and F2 coincide with H1 and H2). The shape of the pulse H12 is different in each case due to limitation of bandwidth associated with the 700 µm thick crystals. As such, it is observed that the pulse H12 in type-I phase-matching has more bandwidth and is shorter (see Fig. 5b), while the pulse H12 in type-II phase-matching has less bandwidth and is longer (see Fig. 5d). Nevertheless, this result demonstrates the possibility not only to generate but also to isolate the product of time convolution between two optical pulses and, thus, the capability to perform linear filtering operations with optical signals.

## Discussion

In a conventional time-domain SHG process, two pulses separated in time cannot interact with each other since they do not overlap temporally. Thus this operation results in two second harmonic pulses separated by the initial delay. In comparison, when the same pulse pair propagates through the FSHG system exploited here, we showed that the field is processed into three individual second harmonic pulses, as a result of a temporal convolution of the input field with itself. We have evidenced that the central pulse carries the combined phase and amplitude information from both fundamental pulses. This feature is attributed to the instantaneous parametric response of the nonlinear crystal, acting as a dynamic transfer function in this system.

In the case of two pulses delayed by $2\tau$ (see Fig. 1b), the input time-dependent field $E\left(t\right)$ entering the FSHG device is given in complex notation by:

3 $$\mathbf{E}\left(\mathbf{t}\right)=\left|{\mathbf{E}}_{1}\left(\mathbf{t}\right)\right|\mathbf{exp}\mathbf{i}\left({{\varvec{\upomega}}}_{0}\left(\mathbf{t}+{\varvec{\uptau}}\right)+{{\varvec{\upphi}}}_{1}\left(\mathbf{t}\right)\right)+\left|{\mathbf{E}}_{2}\left(\mathbf{t}\right)\right|\mathbf{exp}\mathbf{i}\left({{\varvec{\upomega}}}_{0}\left(\mathbf{t}-{\varvec{\uptau}}\right){+{\varvec{\upphi}}}_{2}\left(\mathbf{t}\right)\right)$$

With the corresponding complex spectral amplitude:

4 $$\stackrel{\sim }{\mathbf{E}}\left({\varvec{\upomega}}\right)=\left|{\stackrel{\sim }{{\varvec{E}}}}_{1}\left({\varvec{\upomega}}\right)\right|\mathbf{exp}\mathbf{i}\left({{\varvec{\Phi}}}_{1}\left({\varvec{\upomega}}\right)-{\varvec{\uptau}}\left({\varvec{\upomega}}-{{\varvec{\upomega}}}_{0}\right)\right)+\left|{\stackrel{\sim }{{\varvec{E}}}}_{2}\left({\varvec{\upomega}}\right)\right|\mathbf{e}\mathbf{x}\mathbf{p}\mathbf{i}({{\varvec{\Phi}}}_{2}\left({\varvec{\upomega}}\right)+{\varvec{\uptau}}\left({\varvec{\upomega}}-{{\varvec{\upomega}}}_{0}\right))$$

For interfering fields, the resulting spectrum is modulated, with a fringe separation of $\Delta \omega$, in the region where both pulses overlap spectrally, as illustrated in Fig. 1d. In the Fourier plane, this corresponds to a spatial modulation of the intensity distribution. Since the second harmonic is generated in this plane, the spatial modulation is preserved in the second-harmonic field, leading to a modulation of $2\Delta \omega$ of the SHG spectra (see Fig. 1e). After recombination to the time-domain, three second harmonic pulses separated by $\tau$ propagate at the output of the FSHG device. They can be understood by developing Eqs. (1) or (2) and considering the sum of two delayed fields ${E}_{1}$ and ${E}_{2}$, which results in three distinct output pulses at twice the frequency (Fig. 1c). For instance, by considering the angular frequency-dependent equations, by substituting Eq. (4) into (1), the second harmonic field can be written as:

5 $$\stackrel{\sim }{\mathbf{E}}\left(2{\varvec{\upomega}}\right)\propto {{\stackrel{\sim }{{\varvec{E}}}}_{1}}^{2}\left({\varvec{\upomega}}\right)+2\cdot {\stackrel{\sim }{{\varvec{E}}}}_{1}\left({\varvec{\upomega}}\right)\cdot {\stackrel{\sim }{{\varvec{E}}}}_{2}\left({\varvec{\upomega}}\right)+{{\stackrel{\sim }{{\varvec{E}}}}_{2}}^{2}\left({\varvec{\upomega}}\right)$$

Equation 5 comprises three terms, likely corresponding to the experimental pulses H1, H12 and H2. From this equation, each term generated at a different delay carries amplitude and phase from the fundamental pulses (F1 and F2 in experiments): ${{\stackrel{\sim }{E}}_{1}}^{2}$ at − $\tau$, a cross-term ${{\stackrel{\sim }{E}}_{1}}^{2}\cdot {{\stackrel{\sim }{E}}_{2}}^{2}$ at zero delay, and ${{\stackrel{\sim }{E}}_{2}}^{2}$ at + $\tau$, see Fig. 1c. Thus, like observed in the experiments, the first and third terms carry the amplitude and phase information from the early and late input pulses, while the middle pulse is carrying the coupled information from both input pulses. As such, the cross-term corresponds to the pulse H12 in the experiments with an amplitude proportional to the product $\left|{\stackrel{\sim }{E}}_{1}\left(\omega \right)\right|\cdot \left|{\stackrel{\sim }{E}}_{2}\left(\omega \right)\right|$ and with a spectral phase ${\Phi }_{1}\left(\omega \right)+{\Phi }_{2}\left(\omega \right)$. For this cross-term, the sum of the spectral phases is cancelling out the linear terms which are opposite. As a consequence, this pulse is generated midway between the two initial pulses on the time axis of the optical field.

To explain the results of polarization dependence shown in Fig. 5, Eq. (5) can be re-written to include the control from the polarization states of F1 and F2:

6 $$\stackrel{\sim }{\mathbf{E}}\left(2{\varvec{\upomega}}\right)\propto {{\stackrel{\sim }{{\varvec{E}}}}_{\parallel }}^{2}\left({\varvec{\upomega}}\right)+2\cdot {\stackrel{\sim }{{\varvec{E}}}}_{\parallel }\left({\varvec{\upomega}}\right)\cdot {\stackrel{\sim }{{\varvec{E}}}}_{\perp }\left({\varvec{\upomega}}\right)+{{\stackrel{\sim }{{\varvec{E}}}}_{\perp }}^{2}\left({\varvec{\upomega}}\right)$$

From this equation, the amplitude and phase should also be transferred linearly to the different terms, although it was not proven experimentally due to the use of thick crystals. In this case, the important observation is that only the cross-term of ${\stackrel{\sim }{E}}_{\parallel }\left(\omega \right)$ and ${\stackrel{\sim }{E}}_{\perp }\left(\omega \right)$ can be generated from SHG in the type II phase-matching configuration like observed in Fig. 5d.

Since this central waveform itself is the result of a temporal convolution between the two input pulses, it implies that its amplitude and phase can be controlled linearly with any of the input pulses. This time convolution leads to far-reaching consequences: If we assume a pulse ${{\varvec{E}}}_{1}\left({\varvec{t}}\right)$ of constant amplitude and phase, interacting through this FSHG system with an arbitrary pulse delayed in time ${{\varvec{E}}}_{2}\left({\varvec{t}}+\Delta {\varvec{t}}\right)$ which spectrally overlaps with ${{\varvec{E}}}_{1}$, a copy of the frequency doubled pulse ${{\varvec{E}}}_{2}$ leaving the system at a different time would be generated. Noticeably, for an external observer positioned after the FSHG system, this copy would leave the system *before* the original pulse, even though both pulses propagate the same distance.

The time window covered by the convolution is not infinite and is defined mainly from the spectral resolution of the system, which is related to the geometry of the Fourier plane. In this projection, all frequencies are spectrally separated and focussed and each focus consists of a narrow spectral slice corresponding to the spectral resolution of the *4f*-setup. Typically, the transform-limited pulse duration of each spectral slice is of several picoseconds. Thus, the two fundamental pulses can interact as long as they are separated by a delay shorter than this characteristic duration. This condition is met naturally if the *4f*-design offers sufficient spectral resolution. For example, if the fringe separation of a modulated spectrum is resolved spatially in the Fourier plane, the interaction between two delayed pulses is enabled (see Fig. S3 in Supplementary Information). In our experiment, we observed that beyond a 4 ps delay between F1 and F2, the convolved signal H12 was decreasing, in agreement with the time window offered by the spectral resolution of system (see equation S1 in Supplementary Information).

As shown by the results, we were able to generate a second-harmonic pulse, advanced in time, that carries the amplitude and phase information of an input pulse arriving at a later time. This is possible because the employed nonlinear crystal, in this optical system, plays the role of a dynamic filter oscillating at optical frequencies. This filter adapts the transfer function of the system in real-time through its nonlinear response.

In conclusion, through the mutual interaction of two femtosecond pulses separated in time by several picoseconds, we have demonstrated that an optical system using second harmonic generation in the frequency-domain can be used as a time operator in optical signal processing. The temporal convolution is the function that defines the interaction between an input signal (e.g., a pulse) and any system that operates as a linear filter, i.e. such that the output signal is given by the temporal convolution between the input signal and the system’s temporal impulse response. In our experiments we have implemented an all-optical convolution operation in which the transfer function of the system is adapting dynamically to the input field through the instantaneous response of the nonlinear medium. This way, we were able to transfer information within the delay axis of an ultrashort laser field in real-time. The proposed concept would thus enable temporal convolution of consecutively incoming signals (e.g. two consecutive optical pulses, but also sequences of pulses), opening unprecedented opportunities for rapid manipulation of these signals, given that the system offers instantaneous optical response. Such an operation could find important applications for pulse shaping in the UV/VIS spectral range and thus coherent control experiments in atomic, molecular and optical (AMO) and condensed matter physics. Fully customized pulses, changing on high repetition rates, can be generated through the control of fundamental input pulses.

## Materials and methods

The experiments were performed using the 2.5 kHz Titanium-Sapphire laser system of the Advanced Laser Light Source (ALLS) laboratory that provides pulses of 35 fs duration and centered at 780 nm. The beam is sent to a Michelson interferometer to create a pulse pair with variable delay. The Michelson is made of a first 50/50 beam splitter to separate the beam in two arms. In one of the Michelson arms, a manual translation stage allows to adjust the relative delay between the two pulse replicas. After following different optical paths, a second identical beam splitter is used to recombine the two pulses in a co-propagating direction. Then, the pulse pair after the Michelson interferometer is frequency doubled through a frequency-domain second harmonic generation (FSHG) system employing a Beta Barium Borate (BBO) crystal positioned in the frequency plane. The *4f*-setup used for FSHG is made using two gratings with 600 lines/mm groove density and two focusing optics with 250 mm focal length. A type I BBO crystal whose thickness is 150 µm is placed in the Fourier plane. Note that for the type-I versus type-II experiment, 700-µm thickness BBO crystals were used instead. In the second half of the FSHG setup, a spherical mirror is used instead of a lens to avoid chromatic dispersion between the pulses at 790 and 395 nm. After the second grating, the fundamental and the second harmonic beams are propagating collinearly along their first and second order of diffraction, respectively. At the input of the FSHG setup, the total pulse energy is 100 µJ. At the output, the energy at 790 nm is 46 µJ and the converted energy at 395 nm is 1.2 µJ. As a reference ultrashort pulse, a small fraction of the fundamental pulse at 790 nm is selected at the laser output to characterize all the pulses (fundamental and second harmonic) at the output of the FSHG setup performing XFROG measurements^10^. The XFROG spectrograms are obtained by sum-frequency mixing between the reference pulse and the output pulses in a BBO crystal whose thickness is 25 µm. The crystal orientation is adjusted either for the characterization of the fundamental or the harmonic pulses, and the signals are measured near 395 nm and 263 nm respectively. This way, the measurements between the fundamental and harmonic pulses are referenced with an absolute delay since the optical path of the reference pulse is fixed. The sum-frequency signals after the XFROG crystal are emitted in different directions but are collected by the same lens and are coupled to a grating based spectrometer (HR4000, Ocean Optics). More details on the experimental layout are provided in the Supplementary Information document.

## Supplementary information

Supplementary file1
