# Supplementary material for: Information transfer via temporal convolution in nonlinear optics
Source: Sci Rep. 2020 Sep 11;10:14969. doi: 10.1038/s41598-020-72170-9 (PMC7486391; doi:10.1038/s41598-020-72170-9)
Supplement: Supplementary file 1 — Supplementary file1 [file 41598_2020_72170_MOESM1_ESM.docx]

Information transfer via temporal convolution in nonlinear optics : supplementary information

Philippe Lassonde,^1,*^ Heide Ibrahim,^1^ Adrien Leblanc,^1^ José Azaña,^1^ Bruno E. Schmidt,^2,*^ François Légaré^1^

^1^INRS-émt, 1650 blvd Lionel-Boulet, Varennes, J3X1S2, QC, Canada

^2^ Few-cycle inc., 2890 rue de Beaurivage, Montreal, H1L5W5, QC, Canada

^*^Corresponding authors: [lassonde@emt.inrs.ca](mailto:lassonde@emt.inrs.ca); [schmidtb@few-cycle.com](mailto:schmidtb@few-cycle.com)

This document provides supplementary information to “Information transfer via temporal convolution in nonlinear optics.” It includes more details on the optical layout used for the main experiment consisting in frequency doubling two delayed pulses in the Fourier plane of a pulse shaper in a 4f geometry. We provide spatial characterization of the beam in the Fourier plane and the farfield distributions of both fundamental and second harmonic fields at the output of the system. Also, we provide results of a benchmark test validating the contribution from the fundamental field to the second harmonic pulses generated at different delays. For this, we compare the second harmonic output generated from interferometric and non-interferometric input fields.

**1. Experimental setup**

The experiments were performed using the 2.5-kHz Titanium-Sapphire laser system of the Advanced Laser Light Source laboratory (INRS-EMT), which provides pulses of 35 fs duration, centered at 790 nm. Pulse pairs with a controllable delay were generated in a Michelson interferometer. A schematic of the experiment is shown in figure S1. They are frequency doubled by propagating through a FSHG setup after which all pulses (fundamental and second harmonic) are characterized through XFROG measurements ^1^.

**
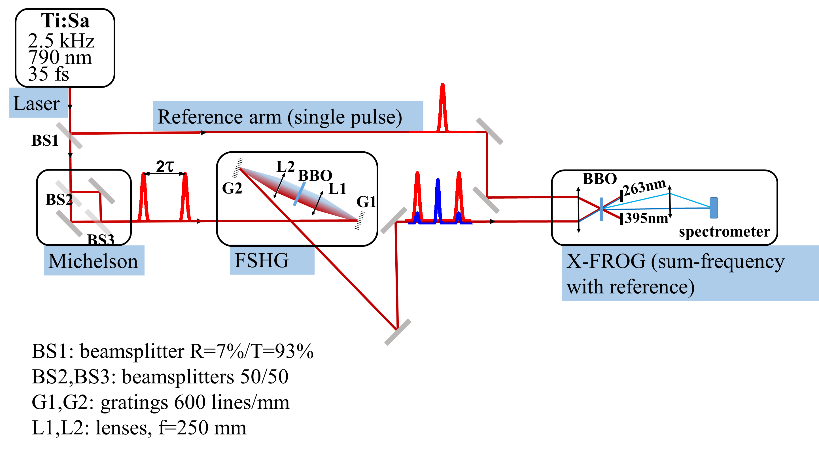
**

Fig. S1. Optical layout of the interferometric FSHG experiment. The laser is a Ti:Sa chirped pulse amplification system. The experiment is depicted in modules comprising a Michelson interferometer, a FSHG frequency conversion stage and the XFROG using a reference ultrashort pulse at 790 nm.

**2. Spatial characterization of the fields**

The optical system is projecting the spectrum of the laser field after the focus of the first lens. Like in a pulse shaper, the input beam is diffracted by the first grating, which creates angular wavelength dispersion. Then, the first lens is collimating the directions of the separated wavelength components so that individual beams are propagating parallel beside each other after the lens. At the focus of the lens, the spectrum is dispersed spatially into individual focal spots spread along the direction of dispersion of the grating. The superposition of the focal spots form a focused line with intensity distributions shown in figure S2. The intensity was measured with a CCD camera. It corresponds to the fundamental field’s spectral distribution centered at 790 nm. In figure S2, it can be observed that the focal spot size is actually defining a spectral resolution of the optical system. It is visible that the spectral interference characteristic of two delayed pulses is better resolved as the thickness of the focused line gets narrower.

Concerning the design of the Fourier plane, the grating and the numerical aperture of the lens are the main parameters influencing the spectral resolution of the Fourier plane. The numerical aperture of the system is the ratio between the input beam diameter and the focal distance of the lens. Therefore, by increasing the input beam diameter, the spectral resolution can be increased like shown in figures S2 and S3. If one is considering to use a different 4f geometry, increasing the grating groove density is increasing the spectral resolution by dispersing the spectrum over a larger area. However, only changing the focal distance has no effect because the distance between the grating and the lens is the focal distance so that the amount of wavelength dispersion varies in the same proportion as the focal spot size. Changing the input beam diameter is actually the only accessible control for a given geometry in order to modify the spectral resolution.


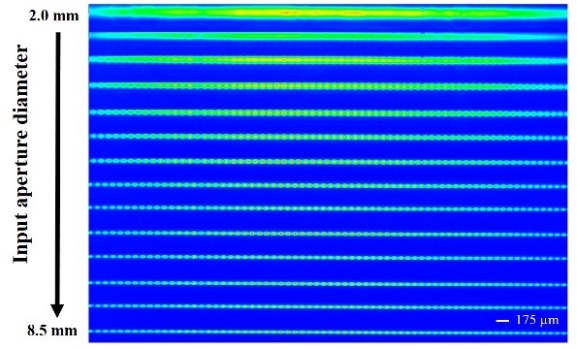


Fig. S2. Evolution of the spatial distribution in the Fourier plane by changing the numerical aperture of the optical system. The intensity distribution is shown as a function of the input beam aperture size varying from 2.0 mm to 8.5 mm by increment of 0.5 mm. As the input beam diameter is increased, the characteristic focal spot size of the system decreases and the spectrum interference is spatially resolved.

In turn, the spectral resolution is inversely proportional to the temporal distribution of the field in the Fourier plane ${\Delta\tau}_{FP}$. In our experiment, this feature can be assumed as the Fourier plane coherence time, which is the upper limit for the delay between the two pulses. Beyond this coherence time, the spectral resolution becomes insufficient and the middle second harmonic pulse is vanishing gradually for larger time separations. This coherence time is deduced from the ratio between the focal spot size, $\Delta y$, and the dimension of the spectrum on the dispersion axis, $\Delta x$, according to the following relations:

$\frac{\boldsymbol{\Delta}\boldsymbol{x}}{\boldsymbol{\Delta}\boldsymbol{y}}\boldsymbol{\propto}\frac{{\boldsymbol{\Delta}\boldsymbol{\lambda}}_{\boldsymbol{spectrum}}}{{\boldsymbol{\Delta}\boldsymbol{\lambda}}_{\boldsymbol{focus}}}\boldsymbol{\propto}\frac{{\boldsymbol{\Delta}\boldsymbol{\tau}}_{\boldsymbol{FP}}}{{\boldsymbol{\Delta}\boldsymbol{\tau}}_{\boldsymbol{TL}}}$ **(S1)**

In equation (S1), ${\Delta\lambda}_{spectrum}$ represents the full bandwidth of the laser, and ${\Delta\lambda}_{focus}$, the bandwidth covering the size of one focal spot. The minimum pulse duration is the Fourier transform limit of the laser spectrum, ${\Delta\tau}_{TL}$. For example in figure S3 (a)-(c), the focal spot size varies from 200 microns to 55 microns. On the other axis (figure S3 (d)-(f)), the spectrum extends over 6.5 mm. From the Fourier-transform limit duration of the full spectrum, 35 fs for this laser, it is straightforward to estimate the duration of the field in the Fourier plane. In our geometry, the maximum coherence time is estimated to 4.1 ps for the larger aperture diameter of 8.5 mm.


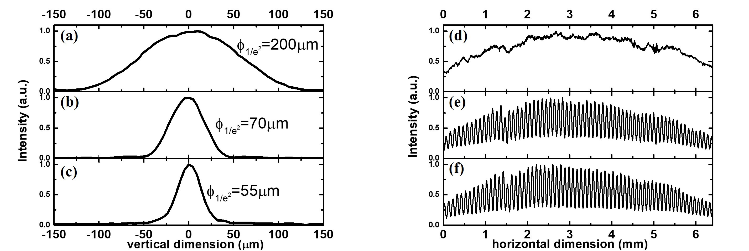


Fig. S3. Spatial and frequency resolution of the Fourier plane. 1-D intensity profiles taken at the center of the Fourier plane distribution in both axis; (a)-(c) vertical and (d)-(f) horizontal; a diaphragm is positioned at the input with variable aperture size clipping the beam: (a) and (d) aperture diameter = 2mm, (b) and (e) = 5mm, (c) and (f) = 8.5 mm. With increasing numerical aperture the focal spot size is decreased and the spectral resolution is increased. The spectrum modulation can be observed in (e) and (f). With the pulses delayed by 3.2 ps, the fringe separation is 95 µm.

After the second grating of the 4f setup, the total field is recombined in a single beam. Figure S4 is showing the farfield intensity distribution following the FSHG stage for both the fundamental (figure S4 (a)) and second harmonic fields (figure S4 (b)) propagating on the same optical axis. It was measured with a CCD camera at the focus of a 1 m lens. The distributions are regular with no evidence of spatial chirp. It was verified that the beams at 790 nm and 395 nm are focusing exactly at the same position.


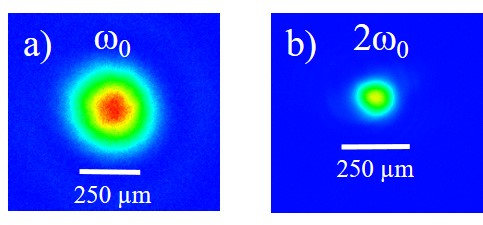


Fig. S4. Farfield distribution after 1m lens. Focal spot was measured at the same position after the FSHG system for (a): the fundamental beam at 790 nm and (b): the second harmonic beam at 395 nm. For (a), the nonlinear crystal was removed from the Fourier plane. For (b), a filter (BG39) was used to filter out the fundamental beam.

**3. Benchmark experiment**

A simple test is made to validate the respective contribution from the fundamental pulses to the generated second harmonic pulses. Figure S5 is showing that the middle second harmonic pulse can be generated only when the two fundamental pulses are interfering in the Fourier plane. In the top panel (figure S5 (a)), the two pulses are generating three second harmonic pulses at different delays from the temporal convolution of the input field. If one arm of the Michelson interferometer is physically blocked to let only one fundamental pulse going through the system, the central pulse is absent. In figure S5 (b), the most delayed fundamental is blocked while in figure S5 (c), we are blocking the first fundamental pulse instead. In the two later situations, the middle second harmonic pulse disappears.


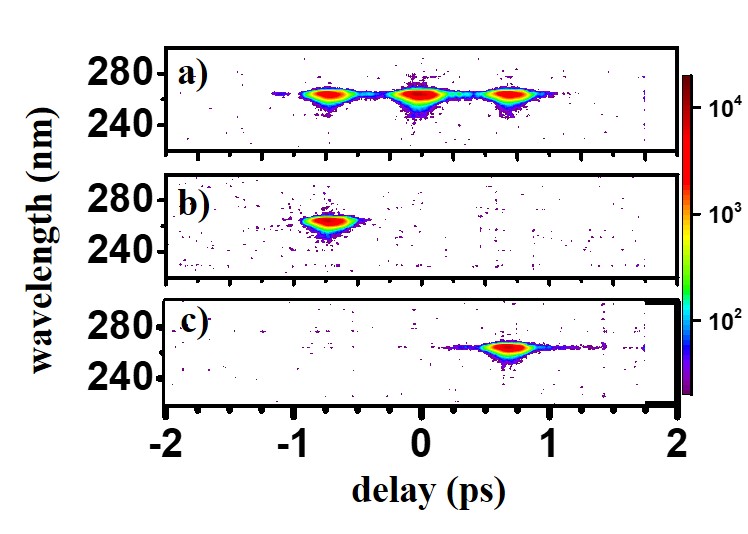


Fig. S5. XFROG traces of the second harmonic field, each for a different situation. In (a), three second harmonic pulses at different delays are generated from two fundamental pulses at 790nm delayed by 1.5 ps and frequency doubled through the FSHG system. In (b), only the first second harmonic pulse is generated if the most delayed fundamental pulse is turned off. In (c), only the last second harmonic pulse is generated if the early fundamental pulse is turned off in turn.

**Reference**

1 Linden, S., Giessen, H. & Kuhl, J. XFROG — A New Method for Amplitude and Phase Characterization of Weak Ultrashort Pulses. *physica status solidi (b)* **206**, 119-124, doi:10.1002/(sici)1521-3951(199803)206:1<119::aid-pssb119>3.0.co;2-x (1998).
